# Corpus Callosotomy in LIS1 Lissencephaly: Postoperative Ketogenic Diet-Related Metabolic Derangements and Feeding Intolerance

**DOI:** 10.7759/cureus.110799

**Published:** 2026-06-13

**Authors:** Abdullah Jalal, Ryan Waggoner, Jimmy Lau, Andrew J George, Hany Salman, Bassel Salman

**Affiliations:** 1 Medicine, Oakland University William Beaumont School of Medicine, Rochester, USA; 2 Medicine, Oakland University William Beaumont School of Medicine, Rochester Hills, USA; 3 Foundational Medical Studies, Oakland University, Rochester, USA; 4 Pediatrics, Corewell Health William Beaumont University Hospital, Royal Oak, USA

**Keywords:** corpus callosotomy, ketogenic diet, lissencephaly, pafah1b1, pediatric epilepsy, vagus nerve stimulation

## Abstract

Classic lissencephaly from *PAFAH1B1* (*LIS1*) haploinsufficiency presents with early, drug-resistant epilepsy; palliative strategies such as corpus callosotomy (CC) and vagus nerve stimulation (VNS) can be effective but are challenging to coordinate in children maintained on ketogenic dietary therapy (KDT). We report the case of a 6-year-old girl with de novo *PAFAH1B1 *deletion who underwent total CC with VNS generator replacement for medically refractory epileptic spasms and focal seizures. Her post-operative course featured early breakthrough seizures responsive to benzodiazepine rescue, then a pediatric ICU (PICU) readmission for emesis, somnolence, edema, and nitrite-positive urinalysis; empiric ceftriaxone was administered while a pretreated catheterized urine culture returned commensal flora/mixed growth, a common interpretive challenge in neurogenic bladder. KDT-associated derangements included β-hydroxybutyrate 8.52 mmol/L, bicarbonate 9-16 mmol/L, and hypoglycemia to 48 mg/dL, managed with acetate-containing IV fluids, protocolized glucose rescue, and ratio adjustment. After a negative anatomic evaluation, erythromycin 75 mg QID improved tolerance to continuous peptide feeds, with near-resolution of emesis by discharge. This case highlights integrated CC+VNS care within KDT using proactive metabolic surveillance, stepwise dysmotility management, and antimicrobial stewardship in neurogenic bladder.

## Introduction

Classic lissencephaly (agyria-pachygyria spectrum) most commonly arises from *PAFAH1B1 *(*LIS1*) haploinsufficiency and manifests with severe cortical malformation, global developmental delay, and refractory epilepsy with epileptic spasms [1,2]. Curative resection is rarely feasible; corpus callosotomy (CC) and vagus nerve stimulation (VNS) reduce disabling seizures and drop attacks in appropriately selected pediatric patients [3,4]. Ketogenic dietary therapy is frequently used in children with drug‑resistant epilepsy but poses unique challenges during neurosurgical admissions, including risks of hypoglycemia, metabolic acidosis, and feeding intolerance [5,6]. There is limited practical guidance on integrating total corpus callosotomy and vagus nerve stimulation generator replacement with ongoing ketogenic therapy in medically fragile children with *LIS1*‑associated lissencephaly. The aim of this report is to describe the peri‑operative seizure, metabolic, and feeding course in such a patient, emphasizing multidisciplinary strategies to maintain ketogenic therapy while managing complications.

## Case presentation

A 6‑year‑old girl with classic lissencephaly had global developmental delay, spastic quadriplegic cerebral palsy, neurogenic bladder (clean intermittent catheterization ×4/day with gentamicin instillations), G-tube dependence, and medically refractory epilepsy despite multi-agent therapy, VNS (12/2022), and home KDT at 1.75:1. She was non‑ambulatory, non‑verbal, and dependent for activities of daily living. She was born at term after a breech pregnancy that was externally rotated prior to vaginal delivery; the neonatal course was uncomplicated, and she did not require NICU care. Her parents were white. There was no family history of seizures, epilepsy, major genetic or neuromuscular disorders, or stroke. Subsequent genetic testing demonstrated a *PAFAH1B1 *deletion (exons 3-11) consistent with *LIS1*‑associated classic lissencephaly, along with a separate 902‑kb 17p13.3-p13.2 deletion not involving *YWHAE*.

Her epilepsy began in 2019 with focal seizures arising from the right frontal region, associated with head jerking to the right, bilateral arm jerking, and then convulsions; MRI at that time demonstrated pachygyria/agyria consistent with classic lissencephaly. Over subsequent years, she developed infantile spasms and medically refractory epileptic spasms despite sequential trials of levetiracetam, vigabatrin, topiramate, adrenocorticotropic hormone, valproic acid, clobazam, initial and later re‑initiated ketogenic dietary therapy, and placement of a vagus nerve stimulator in December 2022. By 2024-2025, she had epileptic spasms occurring daily, often in clusters, with intermittent convulsive seizures and frequent hospitalizations despite a 1.75:1 ketogenic diet, vigabatrin, valproic acid, cannabidiol, and levetiracetam; her most recent long‑term video‑EEG demonstrated multiple epileptic spasms and four tonic‑clonic seizures. Table 1 summarizes the clinical timeline.

**Table 1:** Clinical timeline VNS: vagus nerve stimulation; KDT: ketogenic dietary therapy; PACU: post-anesthesia care unit; PICU: pediatric ICU; PICC: peripherally inserted central catheter; CC: corpus callosotomy

Date	Event	Key details
2019	Epilepsy onset	Focal seizures from the right frontal region with head jerking to the right, bilateral arm jerking, and progression to convulsion. MRI showed pachygyria/agyria consistent with classic lissencephaly.
12/2022	VNS placement	Vagus nerve stimulator placed for medically refractory epilepsy.
2024–2025	Preoperative baseline	Daily epileptic spasms, often in clusters, with intermittent convulsive seizures and frequent hospitalizations despite multidrug therapy, KDT, and VNS.
8/18/2025	Total corpus callosotomy + VNS generator replacement	Underwent total corpus callosotomy via right frontal-parietal craniotomy with VNS generator replacement. Early PACU and first-night seizures were aborted with benzodiazepine rescue.
8/28/2025	PICU readmission	Readmitted for emesis, somnolence, and edema. Nitrite-positive urinalysis prompted empiric ceftriaxone.
8/29/2025	PICC placement	PICC placed for access during PICU course.
8/30/2025	Key laboratory evaluation	Marked ketosis, metabolic acidosis, hypoglycemia, and hypoalbuminemia documented.
Early PICU course	KDT-related metabolic derangements	Managed with acetate-containing fluids, glucose rescue, and KDT ratio adjustment.
PICU course	Edema/hypoalbuminemia	Received 25% albumin followed by loop diuretic. Nephrology co-managed acid-base disturbance.
PICU course	Feeding intolerance	Negative anatomic workup; erythromycin 75 mg QID started with continuous peptide feeds. Emesis improved.
9/17/2025	Breakthrough seizure near discharge	A >10-minute seizure terminated with diazepam.
9/18/2025	Discharge	No overnight seizures in the 24 hours before discharge. Discharged on antiseizure medications, erythromycin, pantoprazole, bowel regimen, and continuous peptide feeds.
9/5/2025 MRI	Post-op imaging	MRI showed a small residual splenial remnant along the posterior margin after near-complete callosotomy.
1/7/2026	Outpatient follow-up	Prolonged interval without convulsions after CC; one brief hemiconvulsion aborted by VNS magnet swipe; spasms persisted but no longer clustered.

We obtained a prolonged video-EEG recording, which showed a markedly disorganized, high-amplitude background without posterior dominant rhythm (PDR) or sleep architecture, with abundant diffuse slow spike-wave, multifocal spikes, and generalized/paroxysmal fast activity; multiple epileptic spasms and several tonic-clonic seizures were captured (Figure 1) [2].

**Figure 1:**
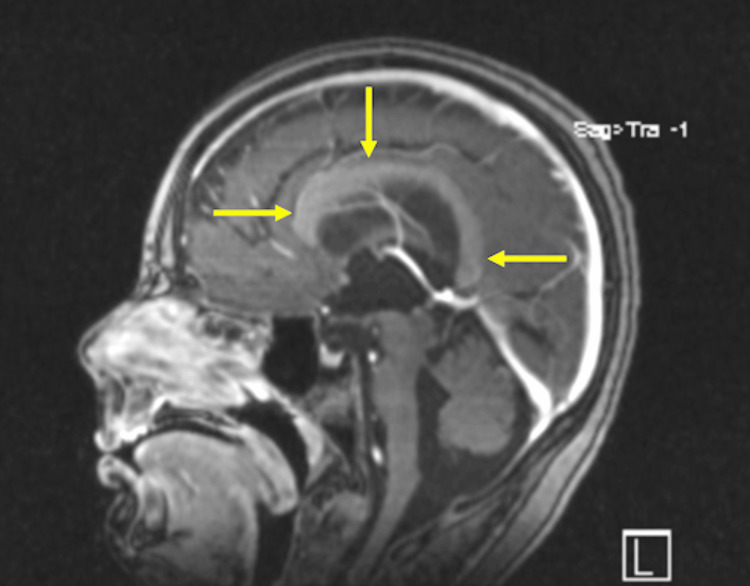
Pre-operative mid-sagittal T1 MRI Diffuse agyria/pachygyria with smooth cortical surface, thickened poorly formed gyri, and ventriculomegaly, consistent with classic lissencephaly; corpus callosum genu, body, and splenium (yellow arrows) are grossly normally developed.

Since she remained medically refractory despite multiple antiseizure medications, vagus nerve stimulation, and ketogenic dietary therapy, and because no resectable focal onset was identified, a multidisciplinary epilepsy team recommended palliative total corpus callosotomy with VNS generator replacement, with the primary goal of reducing disabling convulsive events and drop attacks rather than achieving complete seizure freedom. On 8/18/2025, she underwent total corpus callosotomy via right frontal-parietal craniotomy with VNS generator replacement. Post-operative imaging confirmed expected interhemispheric resection changes (Figure 2).

**Figure 2:**
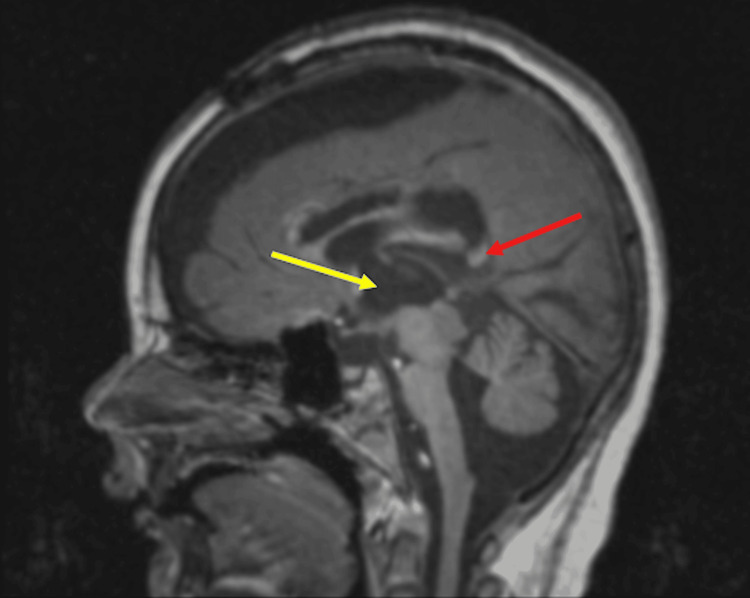
Post-operative mid-sagittal MRI (9/5/2025, without contrast) Interhemispheric resection cavity following total corpus callosotomy (yellow arrow) with small residual splenial remnant along the posterior margin (red arrow). Midline features of classic lissencephaly (smooth cortical surface with thickened, poorly formed gyri) are again present.

Hospital course and management

Immediately post-operatively (8/18/2025), the patient had breakthrough seizures in PACU and overnight that aborted with benzodiazepine rescue; the VNS was returned to prior settings, and she transitioned to floor care. 10 days later, she was readmitted to the PICU for emesis, somnolence, and edema. A catheter urinalysis was nitrite-positive, so empiric ceftriaxone was started while a catheterized urine culture, obtained after >48 hours of antibiotics, reported commensal flora/mixed growth, a known interpretive challenge in neurogenic bladder [7]. A 5-day ceftriaxone course was completed with plans for recollection via catheter when feasible; consistent with stewardship guidance, mixed/commensal growth after recent antibiotics does not establish infection in the absence of compatible symptoms, and repeat catheterized culture is preferred over extending therapy [7]. For access, a peripherally inserted central catheter (PICC) was placed on 8/29 and removed on the day of discharge (9/18).

Ketogenic diet-associated derangements included β-hydroxybutyrate 8.52 mmol/L, high-anion-gap metabolic acidosis (bicarbonate 9-16 mmol/L), and hypoglycemia to 48 mg/dL requiring a bolus of 10% dextrose in water (D10). Management used 0.45% sodium chloride (half-normal saline, **​½**NS)** **with sodium acetate, scheduled glucose/ketone checks with protocolized rescue thresholds, and diet-ratio adjustment under neurology/dietitian guidance (Figure 3) [5,6].

**Figure 3:**
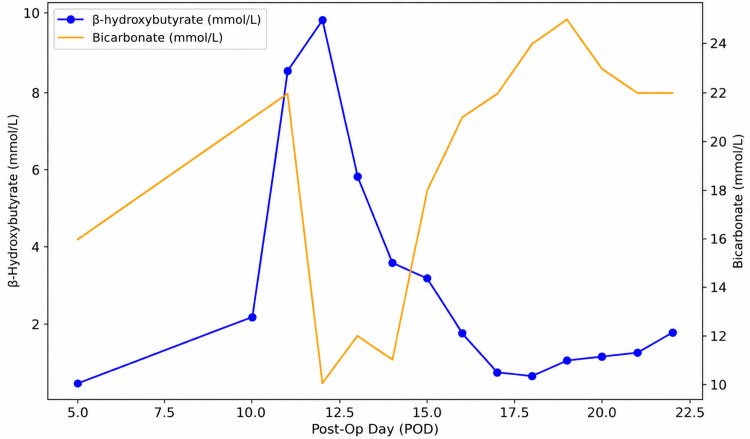
KDT metabolic course by postoperative day β-Hydroxybutyrate (BHB) is plotted as discrete points on isolated draw days (postoperative day (POD) 5 and 10) and as a thin line over the interval with near-daily measurements (POD 12–22); bicarbonate is shown as a daily line on the secondary axis. Vertical reference lines mark key interventions (e.g., acetate-buffered fluids, ketogenic dietary therapy (KDT) ratio change, albumin/diuretic, erythromycin), and markers denote episodes of glucose <60 mg/dL. On days without a BHB measurement, no line is drawn, and the value is not estimated. Graph created using Microsoft Excel (Microsoft 365; Microsoft Corporation, Redmond, USA).

Given edema and hypoalbuminemia during the PICU course, she received 25% albumin followed by a loop diuretic per ICU protocol, and pediatric nephrology co-managed the acid-base disturbances in the context of KDT. Serum albumin was 3.8 g/dL (low) during this period in parallel with the development of edema and improved after albumin and diuretic therapy, whereas creatinine and other renal indices remained within age‑appropriate ranges and inflammatory markers did not suggest systemic infection (Table 2).

**Table 2:** Laboratory values obtained on August 30, 2025 during the PICU course PICU: pediatric ICU; D10: 10% dextrose in water

Laboratory parameter	Value	Interpretation/context
β-hydroxybutyrate	8.52 mmol/L	Marked ketosis during ketogenic diet therapy
Bicarbonate	9–16 mmol/L	High anion gap metabolic acidosis
Glucose	48 mg/dL	Hypoglycemia requiring D10 bolus
Albumin	3.8 g/dL	Low; associated with edema
Creatinine	Within age-appropriate range	No evidence of intrinsic renal dysfunction
Inflammatory markers	Not suggestive of systemic infection	Supported noninfectious explanation for part of the course

Feeding intolerance was evaluated with a negative anatomic workup (prior ultrasound excluding intussusception, normal hepatic/pancreatic labs, and an upper GI series without malrotation/obstruction), favoring dysmotility/gastroesophageal reflux disease (GERD) [8,9]. Enteral erythromycin 75 mg QID (motilin agonist) was initiated alongside continuous peptide formula at 40 mL/h with titrated water flushes, after which emesis frequency declined with near-resolution overnight prior to discharge [8,9]. Interventional Radiology follow-up was arranged to discuss potential gastrojejunostomy (GJ) tubeconversion if vomiting recurred.

Twenty-nine days later, a >10-minute seizure terminated with diazepam; there were no overnight seizures in the 24 hours preceding discharge on 9/18. She was discharged on vigabatrin 1350 mg BID, valproate 650 mg BID, cannabidiol 250 mg BID, levetiracetam 850 mg BID, benzodiazepine rescue per protocol; pantoprazole** **BID, erythromycin 75 mg QID, bowel regimen, and continuous peptide feeds at 40 mL/h [3,10]. For sleep dysregulation, clonidine 50 mcg nightly was continued pending neurology follow-up.

## Discussion

This case illustrates how total corpus callosotomy and vagus nerve stimulation can be integrated within a ketogenic dietary therapy framework for *LIS1*-lissencephaly, provided perioperative risks are anticipated (acid-base surveillance, glucose rescue thresholds, and dysmotility protocols; Figure 3) and device programming is optimized for seizure control (Table 3) [1-3,5-9,10].

**Table 3:** Vagus nerve stimulation parameters at discharge ^†^Duty cycle per manufacturer: (ON + 4 s) / (ON + OFF) = (30+4)/(30+108) ≈ 24.6% → ~25%; ^‡^AutoStim/Magnet inherit the global signal frequency. AutoStim: Automatic stimulation mode that delivers additional vagus nerve stimulation (VNS) in response to device-detected physiologic changes suggestive of a seizure; Magnet: Patient- or caregiver-activated stimulation delivered by swiping the VNS magnet over the generator to abort or shorten seizures.

Mode	Output Current (mA)	Signal Frequency (Hz)	Pulse Width (us)	On-time (s)	Off-time (s)	Duty cycle (%)
Normal (N)	1.75	20	250	30	108	25%^†^
AutoStim (AS)	1.875	20^‡^	250	60	N/A	N/A

The preoperative video-EEG findings, including a markedly disorganized high-amplitude background without a posterior dominant rhythm or sleep architecture and abundant multifocal epileptiform discharges, are consistent with severe epileptic encephalopathy reported in classic lissencephaly [1-2,4]. Systematic reviews favor total over anterior CC for greater seizure reduction, while pediatric VNS frequently achieves ≥50% reduction [3,10]. At outpatient follow-up (1/7/2026), our patient's caregiver reported a prolonged interval without convulsions after CC, with only one brief hemiconvulsion that aborted shortly after VNS magnet swipe; spasms persisted but no longer clustered after CC [3,10]. Before corpus callosotomy, she had epileptic spasms occurring daily, often in clusters, with intermittent convulsive seizures leading to frequent hospitalizations; by follow-up, she had gone several months without generalized convulsions, experienced only rare brief hemiconvulsions aborted by VNS magnet, and had spasms that occurred as isolated events (0-5 per day) rather than in clusters (Table 4).

**Table 4:** Seizure course before and after surgery KDT: ketogenic dietary therapy; VNS: vagus nerve stimulation; PACU: post-anesthesia care unit; CC: corpus callosotomy

Time point	Seizure burden/type	Interpretation
Preoperative baseline	Epileptic spasms occurring daily, often in clusters, with intermittent convulsive seizures and frequent hospitalizations.	Severe drug-resistant epilepsy despite antiseizure medications, KDT, and VNS.
Preoperative long-term video-EEG	Multiple epileptic spasms and four tonic-clonic seizures captured.	Objective confirmation of high seizure burden before surgery.
Immediate postoperative period (8/18/2025)	Breakthrough seizures in PACU and overnight, aborted with benzodiazepine rescue.	Early postoperative seizures persisted but were acutely controlled.
Hospital course before discharge	One >10-minute seizure on 9/17 terminated with diazepam; no overnight seizures in the 24 hours before discharge.	Overall seizure burden improved but was not eliminated.
Outpatient follow-up (1/7/2026)	Prolonged interval without convulsions after CC; one brief hemiconvulsion aborted by VNS magnet swipe; spasms persisted but occurred as isolated events rather than clusters (0–5/day).	Sustained reduction in disabling convulsive seizures, with residual spasms.

This clinical improvement was interpreted alongside the post-operative imaging findings. Post‑operative MRI demonstrated a small residual splenial remnant along the posterior margin. Although the overall appearance was consistent with a near‑complete callosotomy, such residual posterior callosal fibers may partially preserve interhemispheric connectivity and could contribute to persistence of epileptic spasms despite the observed reduction in convulsive seizures. Re‑operation to address this remnant was considered but deferred given the patient’s medical fragility and the meaningful improvement in disabling convulsive events.

In addition to the *PAFAH1B1 *deletion, she carried a separate 902‑kb 17p13.3-p13.2 deletion not involving *YWHAE*. While her cortical malformation and epileptic encephalopathy are most consistent with *LIS1*‑associated classic lissencephaly, this additional deletion may contribute to the overall severity of her neurodevelopmental impairment and comorbidities. However, current gene‑specific data for this region are limited, so we cannot confidently assign specific clinical features to this variant. Beyond these structural and genetic considerations, peri‑operative metabolic management was crucial. KDT guidance anticipates vomiting, hypoglycemia, and metabolic acidosis during illness and supports acetate-containing fluids, protocolized glucose rescue, and ratio adjustments rather than abrupt diet cessation [5,6]. After negative anatomic evaluation, erythromycin is a reasonable motilin-agonist trial to improve gastric emptying and tolerance, acknowledging heterogeneous efficacy [7,8]. In catheterized children with neurogenic bladder, colonization and mixed growth are common; symptom-guided treatment and recollection after pretreatment align with stewardship principles [9,11].

Differential diagnosis (post-operative emesis): anatomic obstruction (excluded), medication effect, KDT-associated ketosis/acidosis with ileus-like dysmotility, and aspiration/GERD; the improvement with erythromycin plus continuous peptide feeds supports a dysmotility-predominant mechanism [5-8].

Limitations

Single-patient experience limits generalizability, and feeding improvement likely reflects combined effects of erythromycin, continuous peptide formula, and convalescence [8,9].

## Conclusions

In this patient with severe *LIS1*-associated lissencephaly, total corpus callosotomy with concurrent VNS generator replacement was associated with a meaningful reduction in disabling convulsive seizures after failure of multiple antiseizure medications and ketogenic dietary therapy. Although epileptic spasms persisted and postoperative MRI showed a small residual splenial remnant, the overall clinical course suggested partial surgical benefit, particularly for convulsive events. The postoperative admission also highlights the need for close multidisciplinary management of ketogenic diet-related metabolic derangements, feeding intolerance, and urinary findings in the setting of neurogenic bladder. More broadly, this case illustrates that effective care in severe developmental epileptic encephalopathies often requires coordinated use of surgery, neuromodulation, dietary therapy, and supportive inpatient protocols.
